# D-index: A New Scoring System in Febrile Neutropenic Patients for Predicting Invasive Fungal Infections

**DOI:** 10.4274/tjh.2014.0070

**Published:** 2016-05-16

**Authors:** Gülden Yılmaz, Belgin Coşkun, Atilla Elhan, Alpay Azap, Hamdi Akan

**Affiliations:** 1 Ankara University Faculty of Medicine, Department of Clinical Microbiology and Infectious Diseases, Ankara, Turkey; 2 Ankara University Faculty of Medicine, Department of Biostatistics, Ankara, Turkey; 3 Ankara University Faculty of Medicine, Department of Hematology, Ankara, Turkey

**Keywords:** Neutropenia, D-index, Cumulative-D-index, Hematological malignancies, Invasive fungal infections

## Abstract

**Objective:**

Neutropenia is a critical risk factor for invasive fungal infections (IFIs). We retrospectively performed this study to assess the performance of the D-index, a new test that combines both the duration and the severity of neutropenia, in predicting IFIs among patients with acute myelogenous leukemia.

**Materials and Methods:**

Fifteen patients with IFIs and 28 patients who did not develop IFIs were enrolled in the study. The D-index was defined as the area over the neutrophil curve, whereas the cumulative-D-index (c-D-index) was the area over the neutrophil curve from the start of neutropenia until the first clinical manifestation of IFI.

**Results:**

The D-index and the c-D-index tended to be significantly higher in patients with IFIs, with medians of 10,150 (range: 4000-22,000) and 5300 (range: 2300-22,200), respectively (p=0.037 and p=0.003, respectively). The receiver operating characteristic analyses showed that there was a cutoff point of 3875 for the D-index in predicting IFI; the sensitivity, specificity, and positive and negative predictive values were 100%, 67.9%, 35.4%, and 100%, respectively. There was also a cutoff point of 4225 for the c-D-index in predicting IFI; the sensitivity, specificity, and positive and negative predictive values for the c-D-index were 93.3%, 71.4%, 36.6%, and 98.4%.

**Conclusion:**

The D-index and especially the c-D-index could be useful tools with high negative predictive value to exclude as well as to predict IFIs in the management of neutropenic patients.

## Abstract (tr)

**Amaç:**

İnvaziv fungal enfeksiyonların (İFE) gelişiminde nötropeni önemli bir risk faktörüdür. Biz bu çalışmayı, akut miyeloid lösemi olup, İFE gelişen hastalarda, nötropeni süresini ve sayısını birlikte değerlendiren yeni bir test olan D-indeks’in performansını ölçmek için geriye dönük olarak yaptık.

**Gereç ve Yöntemler:**

Çalışmaya 50 tane İFE gelişen hasta, 28 tane İFE gelişmeyen hasta alındı. D-indeks nötrofil eğrisinin üzerinde kalan alan olarak, kümülatif-D-indeks (k-D-indeks) ise, nötropeninin ilk başladığı günden İFE’nin belirtilerinin başladığı ilk güne kadar çizilen nötrofil eğrisinin üzerinde kalan alan olarak belirlendi.

**Bulgular:**

D-indeks ve k-D-indeks İFE gelişen hastalarda yüksek olma eğilimindedir. D-indeks ve k-D-indeks için değerler ortalama 10,150 (aralık: 4000-22,000) ve 5300 (aralık: 2300-22,200) olup, sırası ile p=0,037 ve p=0,003 saptandı. Yapılan analizlerde D-indeks için eşik değerin 3875 olup, duyarlılık, özgüllük, pozitif ve negatif prediktif değerleri sırası ile %100, %67,9, %35,4 ve %100 saptandı. k-D-index için eşik değer 4225 olup, duyarlılık, özgüllük, pozitif ve negatif prediktif değeri sırası ile %93,3, %71,4, %36,6 ve %98,4 saptandı.

**Sonuç:**

D-indeks ve özellikle k-D-indeks nötropenik hastaları günlük değerlendirmede kullanılabilecek bir testtir. Negatif prediktif değerinin yüksek olması nedeni ile İFE olan hastaları erken yakalamanın yanı sıra, İFE’yi dışlamak için de etkili bulunmuştur.

## INTRODUCTION

Invasive fungal infections (IFIs) are major life-threatening infections among immunocompromised patients with hematologic malignancies. Although there has been significant progress in the management of febrile neutropenic cancer patients related to increasing protective measures and antifungal agents, neutropenia is still a critical risk factor for IFI. Profound (<100 neutrophils/µL) and prolonged (>10 days) neutropenia is associated with a higher risk of invasive aspergillosis [1,2,3,4,5,6,7].

Several scoring systems have been developed to categorize febrile neutropenic patients into risk groups. These systems usually take neutropenia duration into account. Recently, Portugal et al. developed indexes called the D-index and the cumulative-D-index (c-D-index), which take into account both the duration and the intensity of neutropenia to predict the IFI risk [8]. We performed this study to assess the performance of these new tests in predicting IFIs among patients with acute myelogenous leukemia (AML).

## MATERIALS AND METHODS

### Patients

The Department of Adult Hematology of Ankara University’s Faculty of Medicine, a 56-bed institution, is one of the main regional centers of hematology and bone marrow transplantation in Ankara. Patients with newly diagnosed AML receiving first induction or with relapsed or refractory AML, and who developed neutropenia at this center between March 2011 and March 2012, were included in the study. Among these patients, 15 patients with IFIs and 28 patients who did not develop IFIs were enrolled. We selected controls with the same underlying disease and leukemia status. IFIs were classified as possible, probable, or proven according to the European Organization for Research and Treatment of Cancer/Invasive Fungal Infections Cooperative Group and the National Institute of Allergy and Infectious Diseases Mycoses Study Group (EORTC/MSG) revised criteria [9]. Only the proven and probable cases were included in the study (proven: 2, probable: 13). Clinical and epidemiological data were collected by structured survey forms during daily infectious disease consultation visits. The patients who developed IFIs were compared with controls regarding age, sex, underlying disease, comorbidities, type of chemotherapy, antibacterial and antifungal prophylaxis, mortality rate, duration of neutropenia, profound neutropenia, D-index, and c-D-index. The study was approved by the local ethics committee.

### D-index and Cumulative-D-index Calculation

**Figure 1:**
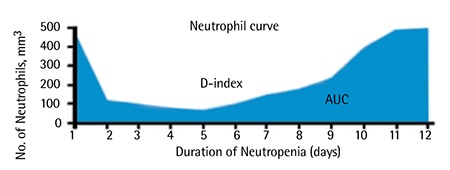
The D-index is an index based on a graph showing absolute neutrophil counts over the course of the episode of neutropenia [8].

The absolute neutrophil count was recorded in patients and controls. The D-index is an index based on a graph showing the absolute neutrophil counts over the course of the episode of neutropenia (Figure 1). It is geometrically the area over the neutrophil curve. The D-index was calculated as the difference between the observed area under curve (AUC) (Ao) and the expected neutrophil area (Ae) if the patient did not develop neutropenia (D-index: Ae-Ao). Ao was calculated by the trapezoidal method, while Ae is the product of 500 and the number of days with neutropenia (Ae: 500/µL x days with neutropenia). An XLA add-in, developed by Usansky et al., was used to apply the trapezoidal method [10].

We also calculated the c-D-index, which is from the start of neutropenia until the date of first clinical manifestation of IFI in patients. The date of first clinical manifestation was defined by 3 specialists (2 from the department of infectious diseases and one from the department of hematology), and then their results were compared with each other. The clinical manifestations were cough, nasal discharge, pleuritic chest pain, hemoptysis, skin nodules, and stomachache with elevated liver enzymes.

### Power Analysis

The D-index was considered as the primary outcome variable for this study. Group sample sizes of 25 and 15 achieved 82% power to detect a difference of 5000 between the null hypothesis that both group means were 4000 and the alternative hypothesis that the mean of group 2 was 9000 with estimated group standard deviations of 5000 and 5000 and with a significance level (alpha) of 0.05 using a 2-sided Mann-Whitney U test, assuming that the actual distribution was normal.

### Statistical Analysis

Mean ± standard deviation, median (minimum-maximum), or percentage values are given as descriptive statistics as applicable. Dichotomous variables were compared using the chi-square or Fisher’s exact test. Test of normality was assessed by Shapiro-Wilk test. Comparison of continuous variables between fungal and control groups was analyzed by Mann-Whitney U test. A receiver operating characteristic (ROC) curve analysis was performed to evaluate the ability of the D-index and c-D-index to predict IFI. Positive and negative predictive values were calculated by using cutoff values obtained from ROC analysis. A multiple logistic regression was performed to identify the independent risk factors of outcome variable and the adjusted odds ratio (OR) was calculated. SPSS 15.0 for Windows was used for statistical analysis. A p-value of less than 0.05 was considered significant.

## RESULTS

A total of 15 patients with IFIs and 28 controls were enrolled during the 1-year study. The clinical and epidemiological data of the patients are shown in Table 1. Those that developed IFIs were older than the controls. The lung was the most common site of fungal infection (86.7%). There were no significant differences between patients and controls regarding sex, status of underlying disease, chemotherapies, and comorbidities. All patients were given fluconazole prophylaxis. The duration and the severity of neutropenia were significantly higher in IFI patients. Consequently, the D-index and the c-D-index tended to be significantly higher in patients with IFIs, with a median of 10,150 (range: 4000-22,000) and 5300 (range: 2300-22,200), respectively.

**Figure 2:**
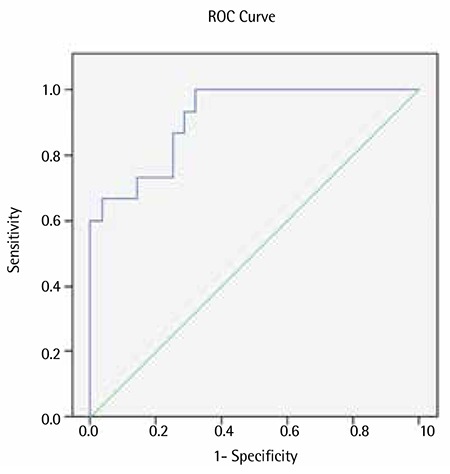
Receiver operating characteristic analyses for the D-index.

**Figure 3:**
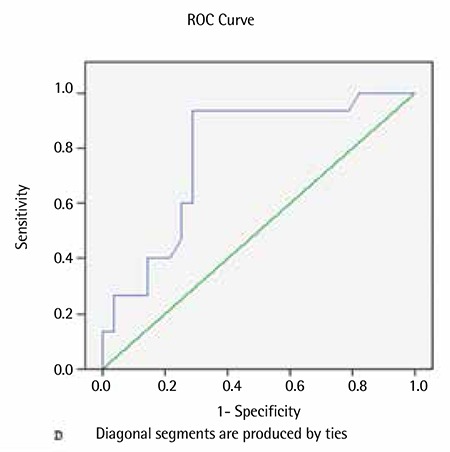
Receiver operating characteristic analyses for the cumulative-D-index.

The ROC analyses showed that both the D-index and the c-D-index could be used to predict IFIs [AUC ± standard error (SE): 0.914±0.042, p<0.001 and AUC ± SE: 0.779±0.074, p=0.003, respectively; Figures 2 and 3]. There was a cutoff point of 3875 for the D-index in predicting IFIs; the sensitivity, specificity, and positive and negative predictive values were 100%, 67.9%, 35.4%, and 100%, respectively. There was also a cutoff point of 4225 for the c-D-index in predicting IFI; the sensitivity, specificity, and positive and negative predictive values for the c-D-index were 93.3%, 71.4%, 36.6%, and 98.4%.

## DISCUSSION

Fungal infections are responsible for most of the deaths from infections in febrile neutropenic patients with hematological malignancies, with mortality rates of 50%-80%. Although the initiation of appropriate antifungal therapy is crucial and associated with improved outcomes, the management of antifungal treatment in this heterogeneous population is a matter of great research [3,6,11,12,13,14].

Empirical antifungal therapy, which is the administration of systemic antifungals for persistent and recurrent fever 96 h after broad-spectrum antibacterial treatment, has been the standard of care for many years. Since the fever-based approach increased antifungal usage, preemptive or diagnostic-driven antifungal therapy, which is usually guided by clinical or radiological signs and serum biomarkers (galactomannan, 1,3-beta-D-glucan, polymerase chain reaction), was defined [15]. Studies comparing these 2 approaches reported that the rate of antifungal usage was reduced and no increase in mortality was observed with diagnostic-driven antifungal therapy. However, the success of this strategy depends on the availability and the performance of the tests predicting IFI [15,16,17,18].

IFIs are difficult to predict and diagnose. Host factors are important for predicting IFIs as well, as they are the determinants of the outcome [6]. Neutropenia, one of the host factors, is still a significant risk factor, and resolution of neutropenia has a key role in complete recovery from an IFI [6,19]. The duration and also the severity of neutropenia are critical, but there was no practical tool that combined both the duration and the severity of neutropenia in its evaluation approach.

Recently, Portugal et al. developed the D-index and c-D-index, simple indexes to calculate, which combine both the duration and the intensity of neutropenia [8]. They reported that these indexes were superior to the duration of neutropenia for predicting IFI. Shortly afterwards, Kimura et al. also showed that early pulmonary infections in hematopoietic stem cell transplantation recipients tended to occur in patients with higher D-index and c-D-index scores [20]. In accordance with these results, higher D-index and c-D-index scores were associated with IFIs in our study. We presume that the c-D-index score in particular, available earlier than the D-index score, has the ability to discriminate among patients with the same duration but different severities of neutropenia according to IFI development.

Previous studies documented increased risks for fungal infections in older patients [11,21,22]. In this study, univariate analysis showed that the median age was higher for patients with IFIs than the controls, at 52.5±15.1 and 42.5±14.6 years, respectively. However, the D-index and c-D-index still tended to be significantly higher for the IFI group than the controls when age and sex were adjusted [OR: 1.54, 95% confidence interval (CI): 1.28-2.19].

Although 20% of the stem cell transplantation centers in Turkey use diagnostic-driven approaches, empirical treatment is still the main approach [23]. This means that a significant proportion of patients are receiving antifungal therapy unnecessarily and we need helpful tools to assess the risk of IFI besides chest computed tomography scan and the use of serum biomarkers. The galactomannan test is the only available serum biomarker at our center. It is performed twice weekly but the results are reported with a 1-week delay. Hence, the c-D-index could be integrated with other parameters to promote diagnostic-driven therapy in such centers.

The negative predictive values of the D-index and c-D-index for IFI prevalence of 15% was 100% (95% CI: 89.8-102.0) and 98.4% (95% CI: 87.2-101.6) using the cutoff values of 3875 and 4225, respectively. The high negative predictive values suggest that this new tool should work to exclude invasive fungal infections. Serum biomarkers such as galactomannan and beta-glucan for fungal infections have some false positives. Thus, when interpreting the results in these situations, a c-D-index of less than 4225 supports the false positivity of other biomarkers and suggests that antifungal therapy could be delayed.

This study has some limitations. The first is the small number of patients. Since April 2012, AML patients with induction therapy have started to receive antifungal prophylaxis (posaconazole) regularly at our center and the impact of this prophylaxis could not be assessed in this study. The second limitation is the underlying disease, due to the fact that only patients with AML were evaluated. Thus, we could not investigate the applicability of this new test with other malignancies.

## CONCLUSION

In conclusion, this study confirms that the D-index, and in particular the c-D-index, could be useful tools to exclude as well as to predict IFIs. These cheap and simple tests stand out with high negative predictive values in daily management of neutropenic patients.

## Ethics

Ethics Committee Approval: The study was approved by the local ethics committee, Informed Consent: N/A.

## Figures and Tables

**Table 1:**
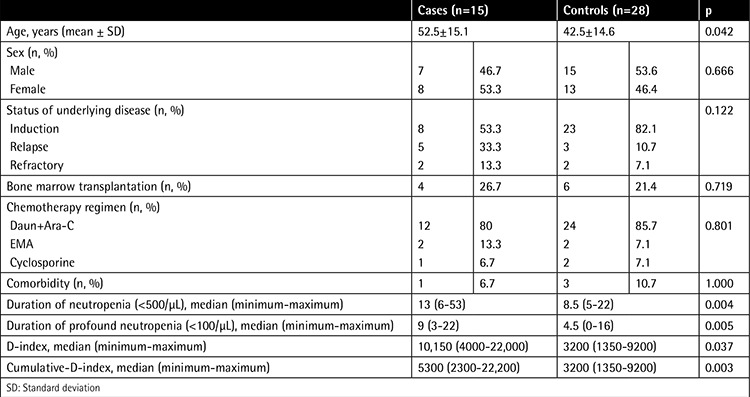
Patient demographics and clinical characteristics.
